# Nonlinear mixed models for characterization of growth trajectory of New Zealand rabbits raised in tropical climate

**DOI:** 10.5713/ab.20.0618

**Published:** 2021-01-01

**Authors:** Vanusa Castro de Sousa, Daniel Biagiotti, José Lindenberg Rocha Sarmento, Luciano Silva Sena, Priscila Alves Barroso, Sued Felipe Lacerda Barjud, Marisa Karen de Sousa Almeida, Natanael Pereira da Silva Santos

**Affiliations:** 1 Graduate Program in Animal Science, Federal University of Piauí, Bom Jesus, PI 64900-000, Brazil; 2 Tecnhical College of Bom Jesus, Federal University of Piauí, Bom Jesus, PI 64900-000, Brazil; 3 Department of Animal Science, Federal University of Piauí, Teresina, PI 64049-550, Brazil; 4 PhD in Animal Science, Federal University of Piauí, Teresina, PI 64049-550, Brazil; 5 Department of Agronomy, Federal University of Piauí, Bom Jesus, PI 64900-000, Brazil

**Keywords:** Absolute Growth Rate, Longitudinal Data, Model Selection, *Oryctolagus cuniculus*, Random Effect

## Abstract

**Objective:**

The identification of nonlinear mixed models that describe the growth trajectory of New Zealand rabbits was performed based on weight records and carcass measures obtained using ultrasonography.

**Methods:**

Phenotypic records of body weight (BW) and loin eye area (LEA) were collected from 66 animals raised in a didactic-productive module of cuniculture located in the southern Piauí state, Brazil. The following nonlinear models were tested considering fixed parameters: Brody, Gompertz, Logistic, Richards, Meloun 1, modified Michaelis-Menten, Santana, and von Bertalanffy. The coefficient of determination (R^2^), mean squared error, percentage of convergence of each model (%C), mean absolute deviation of residuals, Akaike information criterion (AIC), and Bayesian information criterion (BIC) were used to determine the best model. The model that best described the growth trajectory for each trait was also used under the context of mixed models, considering two parameters that admit biological interpretation (*A* and *k*) with random effects.

**Results:**

The von Bertalanffy model was the best fitting model for BW according to the highest value of R^2^ (0.98) and lowest values of AIC (6,675.30) and BIC (6,691.90). For LEA, the Logistic model was the most appropriate due to the results of R^2^ (0.52), AIC (783.90), and BIC (798.40) obtained using this model. The absolute growth rates estimated using the von Bertalanffy and Logistic models for BW and LEA were 21.51g/d and 3.16 cm^2^, respectively. The relative growth rates at the inflection point were 0.028 for BW (von Bertalanffy) and 0.014 for LEA (Logistic).

**Conclusion:**

The von Bertalanffy and Logistic models with random effect at the asymptotic weight are recommended for analysis of ponderal and carcass growth trajectories in New Zealand rabbits. The inclusion of random effects in the asymptotic weight and maturity rate improves the quality of fit in comparison to fixed models.

## INTRODUCTION

In developing countries, rabbit meat is used as an alternative healthy source of protein in the human diet, as this meat is low in calories and has a low fat and sodium content. Furthermore, the consumers’ awareness about the health benefits of consuming rabbit meat has increased significantly [1]. In Brazil, the consumption of this product is still negligible, when compared to other meat categories (e.g., beef, pork, and chicken). Also, the Brazilian consumption of rabbit meat is low in the international context, for example, in comparison to Europe and China [2].

Growth is one of the most important traits used for evaluation of animal production, as a low growth rate may result in low weight at a certain age or delay in slaughter, which would result in animals with low or no market value. For these reasons, studies focusing on growth curves of animals and the development of robust methods of predicting them have increased significantly in animal sciences [3].

Carcass is the main component of live weight and has the highest market value in animals used for meat production. Therefore, muscles positively correlated with the animal finishing (e.g., *Longissimus dorsi*) can be good predictors of carcass composition. In this sense, the technique of real-time ultrasonography has been widely used for the evaluation of carcass and body composition, especially in beef cattle [4], small ruminants [5], and pigs [6]. This technique has been relatively less used for the description of growth patterns in rabbits and, thus, there is still much to be done.

For a description of the development and growth of carcass and its components, nonlinear regression models can be used to evaluate strategies that allow the improvement of animal performance, especially regarding the weight gain and feed efficiency. Parameters of nonlinear models estimated for growth of rabbits can provide information for selection for carcass traits associated with the best slaughter age and prediction of productive efficiency indexes.

The lack of scientific studies regarding the use of mixed models and nonlinear regression applied to the description of ponderal and carcass growth trajectories in New Zealand rabbits (*Oryctolagus cuniculus*) makes it impossible to obtain more reliable definitions of the right age for the slaughter of these animals. Therefore, this study aimed to identify nonlinear regression models, under the context of mixed models, to describe the growth trajectory of New Zealand White rabbits based on weight records and carcass measurements obtained using ultrasonography.

## MATERIALS AND METHODS

### Animal care

All the experimental procedures carried out in this study were approved by the Committee on Ethics in the Use of Animals (CEUA) of the Federal University of Piauí, Brazil (protocol number 328/17).

### Experiment animals and sample collection

The data used in this study were collected from 66 male New Zealand White rabbits (*Oryctolagus cuniculus*), between 2018 and 2019. During the experimental period, the animals were raised in a didactic-productive module of cuniculture located at the Technical College of Bom Jesus of the Federal University of Piauí, in the municipality of Bom Jesus, Piauí, Brazil (latitude 9°04′57.8″S, longitude 44°19′36.8″W at an altitude of 277 m a.s.l.).

After weaning (30-d-old), the animals were maintained in individual galvanized steel cages (80 cm long, 75 cm wide, and 67 cm high) each containing a plastic feeder and a plastic drinker. The cages were kept in a place with temperature regulated by fan ventilation. Water was provided *ad libitum* and 150 g/shift/rabbit of pellet commercial ration was offered to the animals (minimum levels ensured: 88% of dry matter; 12% of moisture; 17% of crude protein; 3.37% of ether extract; 15% of crude fiber; 12% of mineral matter; 2% of calcium; 0.75% of total phosphorus; 0.94% of lysine; 0.63% of methionine + cystine; and 2,300 kcal/kg of digestible energy).

Phenotypic records of live body weight (BW) and loin eye area (LEA) collected every seven days from weaning until 150 days of age were used to determine the model that best describes the growth trajectory of the rabbits. All animals used in the study were weighed during the morning shift using a digital scale.

The measurements of LEA were performed using an ultra-sound machine equipped with a linear transducer (probe) with a frequency of 3.5 MHz. Pictures were collected between the 12th and 13th thoracic vertebrae, at the left side of the animal’s body, perpendicular to the long axis of the *Longissimus dorsi* muscle (loin eye). This anatomical region is widely used for the evaluation of finishing and muscling traits in different species.

### Statistical analyses

The traits BW and LEA were calculated as a function of the animal age (days) using each of the following nonlinear models: Brody [7]; von Bertalanffy [8]; Richards [9]; Logistic [10]; Gompertz [11]; Meloun 1 [12]; modified Michaelis-Menten [13]; and Santana [14]. Four possibilities were compared for the evaluated models (Table 1): a model without random effects named as nonlinear fixed effects model; two models with inclusion of random effect; and a model with inclusion of random effect on the parameters *A* and *k* simultaneously.

Firstly, all the models mentioned above were tested considering the parameters as fixed. In this step, we used the following goodness of fit indicators for the tested models: coefficient of determination (R^2^), calculated as the square of the correlation between the predicted and observed values; mean squared error (MSE); the percentage of convergence (%C) of each model; Akaike information criterion (AIC), which is given by *AIC* = −*log*(*L*) + 2*p*, where *log*(*L*) is the logarithm of the likelihood function of the probability density function; Bayesian information criterion (BIC), expressed as *BIC* = −2log(*L*) + *plog*(*n*); and the mean absolute deviation of residuals (MAD), as proposed by Sarmento et al [15]. MAD is given by the following equation:


$$\text{MAD}=\frac{\sum_{i=1}^{n}\left|Y_{i}-{\hat{Y}}_{i}\right|}{n}$$

where: *Y_i_* is the observed value; *Ŷi* is the estimated value; and *n* is the sample size.

It is important to mention that higher values of R^2^ or %C, as well as lower values of MSE, AIC, BIC, or MAD indicate a better model fit. After choosing the model that best described the growth trajectories for LEA and BW of the studied animals, this model was used under the context of mixed models. For this, we considered two parameters that admit biological interpretation (*A* and *k*), individually or combined. In this step, we used the following criteria to determine the best model: AIC; BIC; and the residual variance ( $\sigma_{e}^{2}$). AIC and BIC were used because these criteria use the analysis of residual independence and the degree of parameterization to improve the precision of fitting of the nonlinear models evaluated. All analyses were performed using the SAS software [16].

Under the context of mixed models, considering *y_ij_* as the measurement *j* (BW or LEA) of the individual *i* and *t_ij_* as the age of this animal (days), the regression model has residuals that follow a normal distribution, with mean zero and constant variance $\sigma_{e}^{2}$. The parameters *A* and *k* were considered as random, with normal distribution, whereas *B* was considered as a fixed parameter.

The observations (*y_ij_*) are independent regarding the index *i*, but not with respect to *j*, because at fixing *i*, the measurements (*y_ij_*) are taken longitudinally for a single animal. Therefore, it is necessary to include intra-individual variance components in the model.

Considering $Ai\sim N(A;\sigma_{a}^{2})$ and $Ki\sim N(K;\sigma_{k}^{2})$, where $\sigma_{a}^{2}$ and $\sigma_{k}^{2}$ are variance components of the parameters *A* and *k*; if *M* = (*Ai*; *Ki*) is a vector of random effects and $N^{\prime }=(A,B,K;\sigma_{e}^{2})$ is a vector of fixed effects, we have *f*(*yi*|*ti*, *N*, *Mi*) *ω* (*Mi*;Σ) is the joint probability density function, where $y_{i}^{\prime }=(yi1;\;yi2;:\;:\;:\;;\;yiJi),t_{i}^{\prime }=(ti1;\;ti2;:\;:\;:\;;\;tiJi),\;\Sigma^{\prime }=(\sigma_{a}^{2};\;\sigma_{k}^{2})$, and *ω* is the joint density of *Ai* and *Ki*. The marginal likelihood function is given by $L(N;\Sigma )=\prod_{I-1}^{n}\int f(y_{i}|t_{i},N,M_{i})\omega (M;\Sigma )dM_{i}$.

The estimators for parameters in *N* and Σ are obtained maximizing L(*N*; Σ) in relation to these numbers. The PROC NLMIXED procedure of the SAS software [16] minimizes numerically −L(*N*; Σ) regarding the parameters *N* and Σ, so that the variance-covariance matrix is approximated to the estimators obtained by the inverse of the Hessian matrix.

For the joint analysis, considering simultaneously *A* and *k* as random, the assumptions for these parameters are described as $\left[\begin{array}{c} A_{i}\\ K_{i} \end{array}\right]\sim NM\left(\left[\begin{array}{c} A_{i}\\ K_{i} \end{array}\right],\left[\begin{array}{cc} \sigma_{a}^{2} & \sigma_{a,k}\\ \sigma_{k,a} & \sigma_{k}^{2} \end{array}\right]\right)$, where *σ_a,k_* = *σ_k,a_* is the covariance between *A* and *k*.

For the biological interpretability of the estimates of parameters, we calculated the absolute growth rate (AGR), inflection point (IP), and relative growth rate (RGR) for each selected model. AGR was obtained from the first derivative of the model with respect to time (∂y/∂t). AGR estimates the increase in the animal weight for each time unit *t* (days or months) in the growth trajectory (i.e., the average growth rate of animals in the population). Regarding IP, this indicates the point where the body growth rate of the animal is the maximum (i.e., the point in which the growth switches from a fast phase to a slower phase). RGR, in turn, is the ratio of AGR to the *ŷ_i_* (predicted BW or LEA) of the selected model.

## RESULTS AND DISCUSSION

The variability of BW and LEA tended to decrease with age (Table 2), probably because the management provided higher uniformity as the animals aged. Several reports have shown that the BW of rabbits tends to increase with their age and older rabbits have higher meat incorporation [17,18]. Therefore, it is expected that male rabbits of the same breed submitted to the same nutritional management, as in the present study, have similar weight gain and carcass development over time.

All the studied models converged; however, the Richards model did not estimate the AIC and BIC values for BW, probably due to the parameterization of this model (Table 3). In general, the comparison of the models used in this study is based on the quality of fit and computational difficulties. We used the percentage of convergence considering the maximum number of iterations of 2,000. Thus, the best model was the one that best fit the data and had the most appropriate results for the studied traits. It is important to mention that the higher the number of criteria considered, the most reliable is the indication of which is the best model in each situation [19].

For BW, we observed that the von Bertalanffy model was the most appropriate (Table 3). On the other hand, the Logistic model was the most appropriate for LEA. The results of this type of comparison rely on the dataset used, which allows one to infer that there is not a single model that is the best in all situations, but there is indeed a model appropriate for each case.

Observing the R^2^, it is possible to note that this criterion was not so informative for LEA, as R^2^ values are considered low for adjustment in this case (Table 3). Regarding BW, R^2^ can be considered only to exclude some models (Richards, Meloun 1, modified Michaelis-Menten, and Santana). Therefore, the coefficient of determination is not a good indicator for model selection, because the other models (Brody, Gompertz, Logistic, and von Bertalanffy) also showed high R^2^ values (0.98). Similar values of R^2^ in different models have also been reported by other authors that used this criterion for the selection of models to describe the growth curve in chickens [20,21]. In these cases, it is necessary to use different criteria of adjustment, due to the low power of decision of R^2^ [21].

Our results showed expressive differences in the variability of the estimates of MSE in functions of the models used. As mentioned before, lower MSE values indicate better adjustments of the model in function of the trait evaluated. MAD is another parameter that indicates the quality of fit of the growth curves; however, this parameter must not be considered exclusively [15].

For the von Bertalanffy model, the inclusion of the random effect in $A\;(\sigma_{a}^{2})$ resulted in a reduction of approximately 11% of the AIC and BIC values (Table 4), in comparison to estimates obtained without the inclusion of random effect.

**Figure 1:**
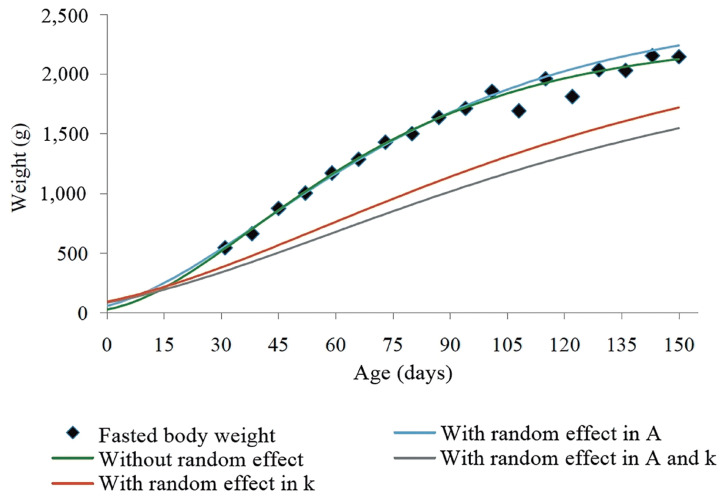
Von Bertalanffy model for description of the ponderal growth trajectory in function of age in New Zealand rabbits. A, asymptotic weight, or average weight at maturity; k, maturity rate.

In the graphical analysis, we can observe that the best adjustment was the one that included the random effect in *A* (Figure 1). On the other hand, the inclusion of two effects in *A* and *k* in the von Bertalanffy model did not show a satisfactory result, probably because the higher number of parameters makes more difficult the convergence of the model, which complicates the estimation of the error.

The parameter *A* represents the estimate of the asymptotic weight, which is interpreted as the adult weight; however, there are controversies about the optimal adult weight, which relies on the species, breed, previous selection, management system, and weather conditions. The parameter *A* indicates the weight that the animal can reach at maturity and is useful for prediction of results and planning of the whole activity regarding the raising and breeding [22,23].

In a study in New Zealand rabbits, Santos et al [24] reported that the Gompertz model had the best fit for growth from birth until 150 days of age. However, the mean adult BW (2,026.84 g) reported by these authors was lower than our findings.

The model selection in the present study did not differ from the findings of Ferreira et al [25], in a study in New Zealand rabbits. In both studies, the von Bertalanffy model had the highest estimate for the parameter *A*.

The parameter *k* represents the growth speed to reach the asymptotic weight (at maturity). In our study, the estimate of *k* obtained using the von Bertalanffy model was 0.01904. Animals with a high maturity rate are considered more precocious than those that have a lower maturity rate. More precocious, prolific, productive, and resistant breeds have a higher market value, as these animals can be slaughtered earlier and with higher carcass yield. This optimizes the costs with feeding (which represents 70% of the total cost of the production) without affecting the environmental and animal welfare rules [26].

With the inclusion of two random effects, the AIC and BIC values also decreased, in comparison to the results obtained without the inclusion of random effect for BW (Table 4). In the dataset used in the present study, the most recommended would be to include only one random effect. This can be justified by the difficulty of convergence of the model, as this model became more parameterized when the two random effects were included. Furthermore, it is important to mention that the lowest residual variance for the von Bertalanffy model was observed when the random effect was included in *A*.

It is important to mention that the parameter *B* is a constant of integration that is related to the initial weight of the animal and represents its degree of maturity at birth. Higher *B* values are associated with lower birth weights in the von Bertalanffy model. In the Logistic growth model, *B* has a fixed value of one; therefore, in this case, there is no biological interpretation [27].

When the random effect ( $\sigma_{a}^{2}$) was included in *A* in the Logistic model used for LEA, the AIC, BIC, and residual variance values decreased by 13.0, 13.5, and approximately 50%, respectively, in comparison to the estimates obtained without the inclusion of random effect (Table 5).

**Figure 2:**
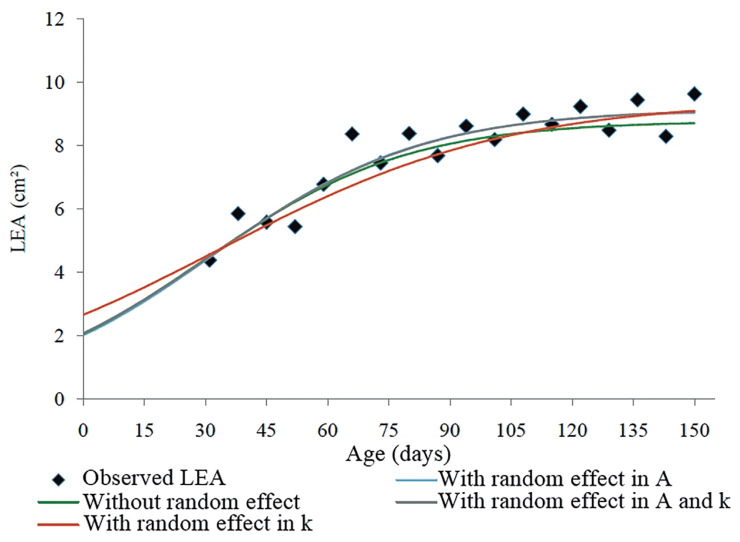
Logistic model including different effects for description of the carcass growth trajectory in function of age in New Zealand rabbits. A, asymptotic weight, or average weight at maturity; k, maturity rate.

Longitudinal data derived from studies on growth may present different variances during an animal’s life. Furthermore, repeated measurements of the same individual generate correlated residuals, which compromise the efficiency of fixed models [28,29]. In the analyses that include random effects in the model, we assumed that the variation in response (BW or LEA) for all individuals follow the same trend (Figure 2); however, the animals may have different behaviors. Random effects represent individual effects as random deviations from the fixed treatment effects and allow each animal to have its own growth trajectory [30].

Regarding the residual variance observed in all models, the use of models containing high residual variance can result in a higher distribution of errors at describing the growth of rabbits.

**Figure 3:**
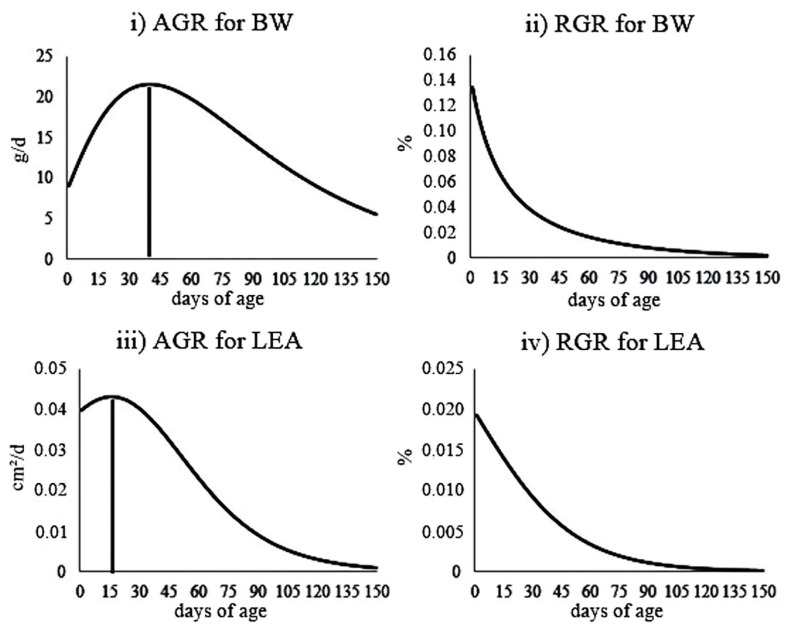
Absolute growth rate (AGR) and relative growth rate (RGR) estimated using the von Bertalanffy (i and ii) and Logistic (iii and iv) models for live body weight (BW) and loin eye area (LEA) in New Zealand rabbits.

The IP for BW was at 40 days of age, when the average BW of the animals was 752.98 g (Figure 3). Therefore, the maximum body growth rate occurred at 40-d-old (21.51 g/d) (i.e., at this time, the growth switched from a fast phase to a slower phase). It is important to mention that the individuals did not stop growing after 40 days of age.

Regarding LEA, the IP was observed at 16-d-old, when the animals had 3.16 cm^2^ of LEA (Figure 3). This indicates that the muscle growth of the studied animals became slower at 16 days of age (0.04 cm^2^/d). This can be justified by the fact that growth has allometric characteristics (i.e., the tissues have different growth rates that change in different phases of the animal life) [17].

The IP was fixed in approximately 30% of the asymptotic weight in the von Bertalanffy model, and approximately 35% of the asymptotic growth of LEA in the Logistic model (Figure 3). Note that the IP of the trajectories coincides with the point of maximum growth rate, which can be useful to define the best moment for slaughter, from the economic point of view. It is important to mention that the age of animals at slaughter is also strongly influenced by the production costs and consumer preferences.

The precocity of ponderal and muscle growths observed in the pre- and post-weaning phases, respectively, indicates that there is a need for adjustments in the feed management of the rabbits after weaning (Figure 3). For the muscle growth at maturity (90-d-old), we observed almost null values (0.003 mm^2^/d). It is important to note that, at this age, the muscle mass reaches its maximum point, thus, the weight gain comes only from gaining fat. Regarding the maturity weight, the animal growth was only 9.09 g/d. From 90 days of age onwards, the weight gain decreased and was lower than that observed at 2-d-old (9.67 g/d). Thus, under conditions similar to those evaluated in this study, it would not be economically advantageous to maintain New Zealand White rabbits in the herd after 90-d-old and animals at this age should be slaughtered for meat production.

The RGR values decreased with age, with estimates that reached 0.8% and 0.1% for BW and LEA using the von Bertalanffy and Logistic models, respectively, at 90 days of age. RGR is expressed as the proportion of the increase in the animal weight or muscle growth for each day with respect to the predicted weight (i.e., the amount of BW that the animal gained in a certain age in relation to its BW at that age). The RGR values in the IP for each trait were 0.028 and 0.014 for BW (von Bertalanffy model) and LEA (Logistic model), respectively (Figure 3). Thus, the gain was proportional to 2.8% and 1.4% for BW and LEA, respectively, in relation to body mass and muscling of the animals.

## CONCLUSION

The von Bertalanffy and Logistic models with random effect at the asymptotic weight are recommended for analysis of ponderal and carcass growth trajectories, respectively, in New Zealand White rabbits. The inclusion of random effects in the asymptotic weight and maturity rate improves the quality of fit in comparison to fixed models.

## Figures and Tables

**Table 1:** Equations of non-linear models including, or not a random effect proposed to explain the growth and carcass trajectories in New Zealand rabbits

Model	Random effect	Equation
Brody [7]	-	***Y*** *_i_* ** = ** ***A*** **(1 − ** ***Be*** **^−^** *^kt^* **) + ** ***ɛ***
	A	***Y*** *_i_* ** = (** ***A*** ** + ** ***u*** **1) (1− ** ***Be*** **^−^** *^kt^* **) + ** ***ɛ***
	k	***Y*** *_i_* ** = ** ***A*** **(1 − ** ***Be*** **^−(^** *^k^* **^+^** *^u^* **^2)^** *^t^* **) + ** ***ɛ***
	A and k	***Y*** *_i_* ** = (** ***A*** ** + ** ***u*** **1) (1 − ** ***Be*** **^−(^** *^k^* **^+^** *^u^* **^2)^** *^t^* **) + ** ***ɛ***
von Bertalanffy [8]	-	***Y*** *_i_* ** = ** ***A*** **(1 − ** ***Be*** **^−^** *^kt^* **)** **^3^** ** + ** ***ɛ***
	A	***Y*** *_i_* ** = (** ***A*** ** + ** ***u*** **1) (1 − ** ***Be*** **^−^** *^kt^* **)** **^3^** ** + ** ***ɛ***
	k	***Y*** *_i_* ** = ** ***A*** **(1 − ** ***Be*** **^−(^** *^k^* **^+^** *^u^* **^2)^** *^t^* **)** **^3^** ** + ** ***ɛ***
	A and k	***Y*** *_i_* ** = (** ***A*** ** + ** ***u*** **1) (1 − ** ***Be*** **^−(^** *^k^* **^+^** *^u^* **^2)^** *^t^* **)** **^3^** ** + ** ***ɛ***
Richards [9]	-	***Y*** *_i_* ** = ** ***A*** **(1 − ** ***Be*** **^−^** *^kt^* **)** *^m^* ** + ** ***ɛ***
	A	***Y*** *_i_* ** = (** ***A*** ** + ** ***u*** **1) (1 − ** ***Be*** **^−^** *^kt^* **)** *^m^* ** + ** ***ɛ***
	k	***Y*** *_i_* ** = ** ***A*** **(1 − ** ***Be*** **^−(^** *^k^* **^+^** *^u^* **^2)^** *^t^* **)** *^m^* ** + ** ***ɛ***
	A and k	***Y*** *_i_* ** = (** ***A*** ** + ** ***u*** **1) (1 − ** ***Be*** **^−(^** *^k^* **^+^** *^u^* **^2)^** *^t^* **)** *^m^* ** + ** ***ɛ***
Logistic [10]	-	***Y*** *_i_* ** = ** ***A*** **(1 + ** ***Be*** **^−^** *^kt^* **)** **^−1^** ** + ** ***ɛ***
	A	***Y*** *_i_* ** = (** ***A*** ** + ** ***u*** **1) (1 + ** ***Be*** **^−^** *^kt^* **)** **^−1^** ** + ** ***ɛ***
	k	***Y*** *_i_* ** = ** ***A*** **(1 + ** ***Be*** **^−(^** *^k^* **^+^** *^u^* **^2)^** *^t^* **)** **^−1^** ** + ** ***ɛ***
	A and k	***Y*** *_i_* ** = (** ***A*** ** + ** ***u*** **1) (1 + ** ***Be*** **^−(^** *^k^* **^+^** *^u^* **^2)^** *^t^* **) ** **^−1^** ** + ** ***ɛ***
Gompertz [11]	-	***Y_i_ = Ae^−e^−B(kt)^^ + ɛ***
	A	***Y_i_ = (A + u1)e^−e^−B(kt)^^ + ɛ***
	k	***Y_i_ = Ae^−^^e^−B((k+u2)t)^^ + ɛ***
	A and k	***Y_i_ = (A + u1)e^−e^−B((k+u2)t)^^ + ɛ***
Meloun I [12]	-	***Y*** *_i_* ** = *A* − *Be*^−^** *^kt^* ** + *ɛ***
	A	***Y*** *_i_* ** = (*A* + *u*1) − *Be*^−^** *^kt^* ** + *ɛ***
	k	***Y*** *_i_* ** = *A* − *Be*^−(^** *^k^* **^+^** *^u^* **^2)^** *^t^* ** + *ɛ***
	A and k	***Y*** *_i_* ** = (*A* + *u*1) − *Be*^−(^** *^k^* **^+^** *^u^* **^2)^** *^t^* ** + *ɛ***
Modified Michaelis-Menten [13]	-	***Y*** *_i_* ** = (*Bk*** *^m^* ** + *At*** *^m^* **) (*k*** *^m^* ** + *t*** *^m^* **)^−1^ + *ɛ***
	A	***Y*** *_i_* ** = [*Bk*** *^m^* ** + (*A* + *u*1)*t*** *^m^* **](*k*** *^m^* ** + *t*** *^m^* **)^−1^ + *ɛ***
	k	***Y*** *_i_* ** = [*B*(*k* + *u*2)** *^m^* ** + *At*** *^m^* **][(*k*+ *u*2)** *^m^* ** + *t*** *^m^* **]^−1^ + *ɛ***
	A and k	***Y*** *_i_* ** = [*B*(*k*+ *u*2)** *^m^* ** + (*A* + *u*1)*t*** *^m^* **][(*k*+ *u*2)** *^m^* ** + *t*** *^m^* **]^−1^ + *ɛ***
Santana [14]	-	***Y*** *_i_* ** = *A*(1 − *e*^−{^** *^kt^* **^[^** *^k^* **^(^** *^t^* **^+^** *^B^* **^)>^−2^^ ) + *ɛ***
	A	***Y*** *_i_* ** = (*A* + *u*1) (1 − *e*^−{^** *^kt^* **^[^** *^k^* **^(^** *^t^* **^+^** *^B^* **^)>^−2^^) + *ɛ***
	k	***Y*** *_i_* ** = *A*(1 − *e*^−{(^** *^k^* **^+^** *^u^* **^2)^** *^t^* **^[(^** *^k^* **^+^** *^u^* **^2) (^** *^t^* **^+^** *^B^* **^)>^−2^^) + *ɛ***
	A and k	***Y*** *_i_* ** = (*A* + *u*1) (1 − *e*^−{(^** *^k^* **^+^** *^u^* **^2)^** *^t^* **^[(^** *^k^* **^+^** *^u^* **^2) (^** *^t^* **^+^** *^B^* **^)>^−2^^ ) + *ɛ***

*Y_i_*, record of animal *i* measured at age *t*; *A*, asymptotic weight or average weight at maturity; *B*, constant of integration; *k*, maturity rate; *m*, shape parameter; *ɛ* error associated with the records; *u*1 and *u*2 are the random effects associated with *A* and *k*, respectively.

**Table 2:** Descriptive statistics of growth and carcass traits of New Zealand rabbits at different ages

Age (d)	Trait	Mean	SD	CV	Minimum	Maximum
31	BW	545.83	176.20	32.28	273.00	987.00
	LEA	4.37	1.08	24.71	1.38	5.96
38	BW	663.24	194.84	29.38	415.00	1,180.00
	LEA	5.84	1.03	17.64	3.24	7.27
45	BW	876.18	200.91	22.93	469.00	1,380.00
	LEA	5.57	0.95	17.06	3.91	7.22
52	BW	1,002.90	234.23	23.36	646.00	1,547.00
	LEA	5.59	1.00	17.89	3.87	6.70
59	BW	1,170.91	221.52	18.92	788.00	1,634.00
	LEA	6.76	1.17	17.31	5.14	9.94
66	BW	1,288.00	228.95	17.78	936.00	1,791.00
	LEA	8.35	1.01	12.10	6.10	9.56
73	BW	1,429.53	253.06	17.70	1,012.00	1,996.00
	LEA	7.44	0.89	11.96	7.17	9.80
80	BW	1,501.90	257.30	17.13	1,095.00	2,052.00
	LEA	8.37	0.42	5.02	7.79	8.80
87	BW	1,637.77	280.41	17.12	1,133.00	2,254.00
	LEA	7.97	0.82	10.29	7.75	8.87
94	BW	1,723.57	304.89	17.69	1,145.00	2,401.00
	LEA	8.60	0.30	3.49	8.06	8.75
101	BW	1,857.24	385.96	20.78	1,130.00	2,560.00
	LEA	8.78	0.28	3.19	8.64	9.42
108	BW	1,692.00	426.22	25.19	1,153.00	2,630.00
	LEA	8.98	0.20	2.23	8.92	9.04
115	BW	1,961.97	395.84	20.18	1,235.00	2,720.00
	LEA	9.66	0.92	9.52	9.01	9.84
122	BW	1,811.15	441.87	24.40	1,250.00	2,830.00
	LEA	9.22	0.11	1.19	9.18	9.26
129	BW	2,037.00	425.67	20.90	1,514.00	3,057.00
	LEA	9.47	0.98	10.35	9.00	9.67
136	BW	2,029.88	425.67	20.97	1,514.00	3,059.00
	LEA	9.46	0.98	10.36	9.00	9.68
143	BW	2,154.00	509.34	23.65	1,656.00	3,219.00
	LEA	8.28	0.91	10.99	6.74	10.08
150	BW	2,147.13	426.51	19.86	1,823.00	3,024.00
	LEA	9.61	0.20	2.08	9.55	9.68

SD, standard deviation; CV, coefficient of variation; BW, body weight (g); LEA, loin eye area (cm^2^).

**Table 3:** Criteria of adjustment of nonlinear regression models in function of carcass and ponderal growth traits in New Zealand rabbits

Model	Criteria of adjustment of the model					

	R^2^	MSE	MAD	%C	AIC	BIC
Live body weight						
Brody	0.98	4,866.57	38.49	81.82	6,675.90	6,692.50
Gompertz	0.98	4,166.72	36.99	87.27	6,675.70	6,692.20
von Bertalanffy	0.98	1,014.84	23.70	90.91	6,675.30	6,691.90
Richards	0.26	280,470.29	384.99	27.27	-	-
Logistic	0.98	5,863.55	46.79	100.00	6,678.50	6,695.10
Santana	0.88	27,390.26	68.78	27.27	6,693.60	6,710.20
Modified Michaelis-Menten	0.04	962,118.38	766.98	34.54	6,683.80	6,704.60
Meloun1	0.32	228,169.88	376.29	90.91	6,676.80	6,693.40
Loin eye area						
Brody	0.38	0.23	0.30	94.50	784.10	798.60
Gompertz	0.47	0.29	0.32	96.36	783.80	798.20
von Bertalanffy	0.43	0.19	0.28	92.73	784.10	798.60
Richards	0.09	3.98	1.39	65.45	1,084.40	1,102.50
Logistic	0.52	0.20	0.28	100.00	783.90	798.20
Santana	0.01	11.33	2.07	49.09	783.80	798.20
Modified Michaelis-Menten	0.01	12.27	2.68	47.27	788.10	806.10
Meloun1	0.29	1.89	0.86	89.09	784.10	798.60

R^2^, coefficient of determination; MSE, mean squared error; MAD, mean absolute deviation; %C, percentage of convergence; AIC, Akaike information criterion; BIC, Bayesian information criterion.

**Table 4:** Von Bertalanffy ponderal growth trajectory parameters in New Zealand rabbits

Model	Parameters		Estimate	SE	95% confidence limit		AIC	BIC
	
	Fixed	Random			Lower	Upper		
I	*A*	-	2,302.13^*^	90.5659	2,124.16	2,480.10	6,675.30	6,691.90
	*B*	-	0.7707^*^	0.06407	0.66448	0.8966		
	*K*	-	0.02255^*^	0.002309	0.01802	0.02709		
	-	$\sigma_{e}^{2}$	95,760.00^*^	6,273.51	83,432.00	108,088.00		
II	*A*	-	2,541.32^*^	83.8496	2,373.21	2,709.43	5,941.2	5,951.2
	*B*	-	0.7158^*^	0.01807	0.6796	0.7521		
	*K*	-	0.01904^*^	0.000822	0.01740	0.02069		
	-	$\sigma_{a}^{2}$	22.5416^*^	0.1275	22.5416	22.5417		
	-	$\sigma_{e}^{2}$	12,359.00^*^	861.90	10,631.00	14,087.00		
III	*A*	-	2,411.63^*^	123.97	2,163.08	2,660.17	6,122.40	6,132.50
	*B*	-	0.6620^*^	0.01013	0.6417	0.6824		
	*K*	-	0.01219^*^	0.000478	0.01123	0.01315		
	-	$\sigma_{k}^{2}$	0.000051^*^	0.00	-	-		
	-	$\sigma_{e}^{2}$	7,465.66^*^	332.97	6,798.09	8,133.22		
IV	*A*	-	2,208.95^*^	191.71	1,824.43	2,593.47	6,145.70	6,159.80
	*B*	-	0.6619^*^	0.01089	0.6400	0.6837		
	*K*	-	0.01185^*^	0.000595	0.01065	0.01304		
	-	$\sigma_{a}^{2}$	38.1235ns	117.26	−197.06	273.31		
	-	*σ* *_a,k_*	0.3499ns	0.4558	−0.5643	1.2641		
	-	$\sigma_{k}^{2}$	0.000077^*^	0.00	-	-		
	-	$\sigma_{e}^{2}$	7,186.80^*^	315.79	6,553.41	7,820.20		

SE, standard error; AIC, Akaike information criterion; BIC, Bayesian information criterion; *A*, asymptotic weight or average weight at maturity; *B*, constant of integration; k, maturity rate; $\sigma_{a}^{2}$, estimate of variance of the asymptotic weight; $\sigma_{k}^{2}$, estimate of variance of the maturity rate; *σ_a,k_*, estimate of covariance between asymptotic weight and maturity rate; $\sigma_{e}^{2}$, estimate of the residual variance.

* Significant at 0.05 probability; ns, non-significant.

**Table 5:** Parameters of the Logistic model for the growth trajectory of loin eye area in New Zealand rabbits

Parameters	Estimate		SE	95% confidence limit		AIC	BIC
	
	Fixed	Random		Lower	Upper		
*A*	-	8.7666^*^	0.1722	8.4276	9.1056	783.9	798.4
*B*	-	3.2755^*^	0.5342	2.2238	4.3272		
*K*	-	0.03995^*^	0.004396	0.03129	0.0486		
-	$\sigma_{e}^{2}$	0.9837^*^	0.08389	0.8186	1.1489		
*A*	-	9.1114^*^	0.2069	8.6934	9.5293	682.1	690.8
*B*	-	3.5473^*^	0.3883	2.7632	4.3314		
*K*	-	0.03942^*^	0.002948	0.03347	0.04537		
-	$\sigma_{a}^{2}$	0.8945^*^	0.2556	0.3783	1.4107		
-	$\sigma_{e}^{2}$	0.4930^*^	0.04635	0.3994	0.5866		
*A*	-	9.4319^*^	0.2149	8.998	9.8658	720.6	729.3
*B*	-	2.5585^*^	0.2257	2.1026	3.0143		
*K*	-	0.02805^*^	0.002355	0.02329	0.0328		
-	$\sigma_{k}^{2}$	0.00005^*^	0.000017	0.000017	0.000085		
-	$\sigma_{e}^{2}$	0.5829^*^	0.05556	0.4707	0.6951		
*A*	-	9.1444^*^	0.1973	8.7457	9.5433	681.5	693.7
*B*	-	3.4151^*^	0.3608	2.686	4.1442		
*K*	-	0.03837^*^	0.00269	0.03293	0.04381		
-	$\sigma_{a}^{2}$	0.8141^*^	0.3138	0.1798	1.4484		
-	σ_a,k_	0.00173ns	0.000974	−0.00024	0.003698		
-	$\sigma_{k}^{2}$	−0.00003^*^	5.1×10−6	−0.00004	−0.00002		
-	$\sigma_{e}^{2}$	0.5304^*^	0.04343	0.4426	0.6181		

SE, standard error; AIC, Akaike information criterion; BIC, Bayesian information criterion; *A*, asymptotic weight or average weight at maturity; *B*, constant of integration; *k*, maturity rate; $\sigma_{e}^{2}$, estimate of the residual variance; $\sigma_{a}^{2}$, estimate of variance of the asymptotic weight; $\sigma_{k}^{2}$, estimate of variance of the maturity rate; *σ_a,k_*, estimate of covariance between asymptotic weight and maturity rate.

* Significant at 0.05 probability; ns, non-significant.

## References

[1] Amer SA, Omar AE, Abd El-Hack ME (2019). Effects of selenium- and chromium-enriched diets on growth performance, lipid profile, and mineral concentration in different tissues of growing rabbits. Biol Trace Elem Res.

[2] Szendrő K, Szabó-Szentgróti E, Szigeti O (2020). Consumers’ attitude to consumption of rabbit meat in eight countries depending on the production method and its purchase form. Foods.

[3] Souza LA, Carneiro PLS, Malhado CHM, Silva FF, Silveira FG (2013). Traditional and alternative nonlinear models for estimating the growth of Morada Nova sheep. R Bras Zootec.

[4] Silva MJFB, Lins LF, Lins NBO (2017). Bovine carcass evaluation: a review about the use of the ultrasound. Med Vet (UFRPE).

[5] Suguisawa L, Marques ACW, Bardi AE, Fausto D (2009). Utilization of ultrasonography as a tool for standardization of commercial carcasses. Tecnol Ciên Agropec.

[6] Jung JH, Shim KS, Na CS, Choe HS (2015). Studies on intramuscular fat percentage in live swine using real-time ultrasound to determine pork quality. Asian-Australas J Anim Sci.

[7] Brody S (1945). Bioenergetics and growth: with special reference to the efficiency complex in domestic animals.

[8] von Bertalanffy L (1957). Quantitative laws in metabolism and growth. Q Rev Biol.

[9] Richards FJ (1959). A flexible growth function for empirical use. J Exp Bot.

[10] Nelder JA (1961). The fitting of a generalization of the logistic curve. Biometrics.

[11] Laird AK (1965). Dynamics of relative growth. Growth.

[12] Meloun M, Militký J (1996). Statistical processing of experimental data: collection of tasks (with diskette).

[13] López S, France J, Gerrits WJ, Dhanoa MS, Humphries DJ, Dijkstra J (2000). A generalized Michaelis-Menten equation for the analysis of growth. J Anim Sci.

[14] Santana TJS (2013). New growth curve models for beef cattle [PhD thesis].

[15] Sarmento JLR, Regazzi AJ, Sousa WH, Torres RA, Breda FC, Menezes GRO (2006). Analysis of the growth curve of Santa Ines sheep. R Bras Zootec.

[16] SAS Institute Inc (2015). SAS/STAT 14.1 user’s guide: The NLMIXED Procedure.

[17] Dalle Zotte A (2002). Perception of rabbit meat quality and major factors influencing the rabbit carcass and meat quality. Livest Prod Sci.

[18] Metzger S, Odermatt M, Szabó A (2011). Effect of age and body weight on carcass traits and meat composition of rabbits. Arch Anim Breed.

[19] Teixeira Neto MR, Cruz JF, Faria HHN, Souza ES, Carneiro PLS, Malhado CHM (2016). Description of Santa Ines sheep growth using non-linear models selected by multivariate analysis. R Bras Saúde Prod Anim.

[20] Mohammed FA (2015). Comparison of three nonlinear functions for describing chicken growth curves. Sci Agric.

[21] Adenaike AS, Akpan U, Udoh JE (2017). Comparative evaluation of growth functions in three broiler strains of Nigerian chickens. Pertanika J Trop Agric Sci.

[22] Carneiro PLS, Malhado CHM, Afonso PRAM (2009). Growth curve in Mambrina goats raised in caatinga. R Bras Saúde Prod Anim.

[23] Teixeira MC, Villarroel AB, Pereira ES, Oliveira SMP, Albuquerque ÍA, Mizubuti IY (2012). Growth curve of lambs from three systems of production in Northeastern Brazil. Semin Cienc Agrar.

[24] Santos DCE, Sousa CA, Silva ES Comparison of non-linear models adjustment in New Zealand rabbits growth curve. http://www.adaltech.com.br/anais/zootecnia2018/resumos/trab-1694.pdf.

[25] Ferreira DSA, Santos ALP, Freitas JR (2019). New non-linear model to describe growth curves of New Zealand rabbits. Sigmae.

[26] Ferreira WM, Machado LC, Jaruche YG (2012). Practical handbook of rabbit production.

[27] Freitas AR (2005). Growth curves in animal production. R Bras Zootec.

[28] Wang Z, Goonewardene LA (2004). The use of MIXED models in the analysis of animal experiments with repeated measures data. Can J Anim Sci.

[29] Ibiapina Neto V, Barbosa FJV, Campelo JEG, Sarmento JLR (2020). Non-linear mixed models in the study of growth of naturalized chickens. R Bras Zootec.

[30] Huang K, Walker CA (2019). Comparisons of statistical models for growth curves from 90-day rat feeding studies. Arch Toxicol.

